# The DYRK Family of Kinases in Cancer: Molecular Functions and Therapeutic Opportunities

**DOI:** 10.3390/cancers12082106

**Published:** 2020-07-29

**Authors:** Jacopo Boni, Carlota Rubio-Perez, Nuria López-Bigas, Cristina Fillat, Susana de la Luna

**Affiliations:** 1 Centre for Genomic Regulation (CRG), The Barcelona Institute of Science and Technology (BIST), Dr Aiguader 88, 08003 Barcelona, Spain; jacopo.boni@crg.eu; 2 Centro de Investigación Biomédica en Red en Enfermedades Raras (CIBERER), 28029 Madrid, Spain; 3 Cancer Science Programme, Institute for Research in Biomedicine (IRB), The Barcelona Institute of Science and Technology (BIST), Baldiri Reixac 10, 08028 Barcelona, Spain; carlotarp@gmail.com (C.R.-P.); nuria.lopez@irbbarcelona.org (N.L.-B.); 4 Institució Catalana de Recerca i Estudis Avançats (ICREA), Passeig Lluís Companys 23, 08010 Barcelona, Spain; 5 Institut d’Investigacions Biomèdiques August Pi i Sunyer (IDIBAPS), Rosselló 149-153, 08036 Barcelona, Spain; cfillat@clinic.cat; 6 Universitat Pompeu Fabra (UPF), Dr Aiguader 88, 08003 Barcelona, Spain

**Keywords:** DYRK kinases, cellular signaling, expression dysregulation, cell cycle, cell survival, tumor progression, kinase inhibitors

## Abstract

DYRK (dual-specificity tyrosine-regulated kinases) are an evolutionary conserved family of protein kinases with members from yeast to humans. In humans, DYRKs are pleiotropic factors that phosphorylate a broad set of proteins involved in many different cellular processes. These include factors that have been associated with all the hallmarks of cancer, from genomic instability to increased proliferation and resistance, programmed cell death, or signaling pathways whose dysfunction is relevant to tumor onset and progression. In accordance with an involvement of DYRK kinases in the regulation of tumorigenic processes, an increasing number of research studies have been published in recent years showing either alterations of DYRK gene expression in tumor samples and/or providing evidence of DYRK-dependent mechanisms that contribute to tumor initiation and/or progression. In the present article, we will review the current understanding of the role of DYRK family members in cancer initiation and progression, providing an overview of the small molecules that act as DYRK inhibitors and discussing the clinical implications and therapeutic opportunities currently available.

## 1. Background

**Figure 1:**
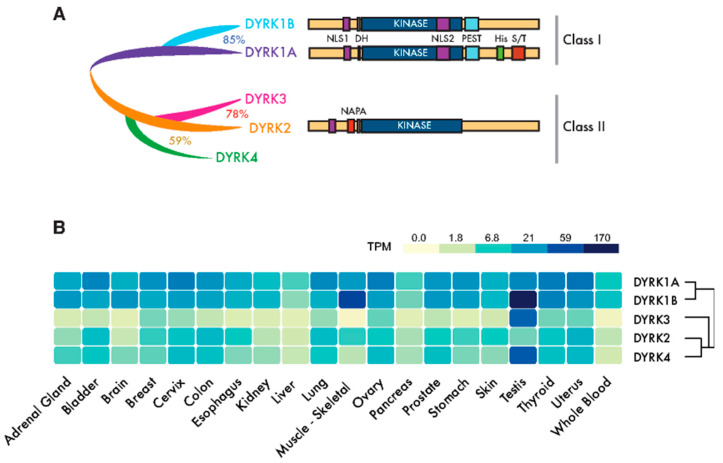
Dual-specificity tyrosine-regulated kinase (DYRK) protein kinases: primary structure and expression. (**A**) Scheme of the mammalian family of DYRKs, indicating their phylogenic relationships, degree of homology and protein domains. The catalytic domain (KINASE) and the DYRK homology box (DH) are common to all members of the family. Class I DYRKs have two nuclear localization signals (NLSs) (NLS1 and NLS2) and a proline-, glutamic acid-, serine- and threonine-rich (PEST) motif. DYRK1A also includes a tract of 13 consecutive histidine residues (His) and a region enriched in serine/threonine residues (S/T) at the C-terminus. Class II DYRKs have a common structure, with the characteristic N-terminal autophosphorylation accessory (NAPA) domain at the N-terminus. In the case of DYRK2 and DYRK4, functional NLSs have been described within the noncatalytic N-terminus. (**B**) The expression of human DYRKs based on the Genotype-Tissue Expression (GTEx) data represented as the median TPMs (transcripts per million: GTEx Analysis Release V8, www.gtexportal.org/home, dbGaP Accession phs000424.v8.p2). Tissues represented in the tumor data in Table S1 were chosen (brain: cortex; cervix: ectocervix; colon: sigmoid colon; esophagus; mucosa, kidney: cortex; skin: suprapubic—not sun-exposed).

The first cancer gene identified, the proto-oncogene *c-Src*, was found to encode a protein kinase [1]. Yet, since then, almost a hundred kinase genes have been attributed a tumor suppressor or oncogenic role, and they represent the most abundant class of cancer driver genes known to date [2]. Dual-specificity tyrosine-regulated kinases (DYRKs) belong to the CMGC group of kinases, which includes cyclin-dependent kinases (CDKs), mitogen-activated protein kinases (MAPKs), CDK-like kinases, the serine-arginine-rich protein kinase, Cdc2-like kinases (CLKs) and members of the RCK family [3]. The DYRK family is formed by three subfamilies: the DYRK subfamily, the homeodomain-interacting kinases (HIPKs), and the pre-messenger RNA-processing protein 4 kinases (PRP4Ks) [3]. Here, we will use “DYRK” to refer specifically to the DYRK subfamily, which contains five members in humans that are clustered into two classes based on their phylogenetic relationships [4]: class I DYRKs, DYRK1A and DYRK1B (also known as Mirk from minibrain-related kinase) and class II DYRKs, DYRK2, DYRK3 (also known as REDK from regulatory erythroid kinase) and DYRK4 (Figure 1A).

DYRK kinases phosphorylate a broad set of substrates that are involved in a wide range of cellular processes, and they are thought to fulfill essential biological functions both during development and in maintaining homeostasis during the adult life. Consequently, the aberrant regulation or expression of DYRK kinases has been associated with several human pathologies, including cancer. In the present article, we will review our understanding of the role of DYRK family members in cancer initiation and progression, providing an overview of the small molecules that act as DYRK inhibitors and discussing the clinical implications and therapeutic opportunities currently available.

## 2. The DYRK Family of Kinases

The members of the DYRK family all share a highly conserved catalytic domain with special features within the CMGC group [5] and the so-called DYRK homology (DH) box motif located upstream of it (Figure 1A). In addition, DYRK kinases present class-specific domains: DYRK1A and DYRK1B harbor a proline-, glutamic acid-, serine- and threonine-rich (PEST) motif in the noncatalytic C-terminal region and equally positioned nuclear localization signals (NLS) (Figure 1A). On the other hand, class II DYRKs present a N-terminal autophosphorylation accessory region (NAPA) domain, essential for catalytic activation [6] (Figure 1A). All human DYRKs accumulate in the cytosol of cells, and DYRK1A, DYRK2 and DYRK4 can be imported into the nucleus by means of dedicated NLSs [7,8,9]. DYRK1A translocation to the nucleus acquires special biological significance, since it has been described as a chromatin-associated kinase capable of regulating the gene expression [10,11], and it is functionally linked to the DNA damage response (DDR) [12,13,14]. Chromatin association in the DDR context has also been recently described for DYRK1B [15]. Moreover, a DYRK1A-specific run of histidine residues targets this family member to the subnuclear splicing compartment [7], and the noncatalytic N-terminal domain of DYRK3 serves to localize it to stress granules [16]. Both the histidine run in DYRK1A and the N-terminus of DYRK3 participate in the generation of phase-separated subcellular compartments [17,18]. Changes in the subcellular localization of DYRK proteins have been observed in response to different signals, such as that of DYRK2 in response to DNA damage or proinflammatory signals [19,20] or DYRK1A in response to Wnt signaling [21]. However, how the subcellular localization of DYRKs is regulated or how it contributes to their activity is still not well-understood.

A high-throughput transcript data analysis indicates that DYRK1A and DYRK1B are expressed ubiquitously in human tissues, whereas class II DYRKs are generally expressed more weakly and in a more tissue-restricted pattern (Figure 1B). The expression of DYRKs is regulated through alternative promoters that generate transcripts with distinct 5′-untranslated regions and/or encoding different N-terminal regions [4]. In addition, alternative splicing generates multiple protein isoforms of unclear functional significance [4,8,22,23,24]. DYRKs are also subject to other post-transcriptional events, such as microRNAs (miR)-mediated gene silencing [25,26,27] or local translation [28].

DYRK kinases are “dual specificity” kinases, as they can phosphorylate both tyrosine (Y) and serine/threonine (S/T) residues, although Y-phosphorylation is limited to their autophosphorylation activity [29]. These kinases are activated by the phosphorylation of residues within the activation loop, which drives a conformational switch from the inactive to active state [30,31]. Unlike other kinase families, this key event in DYRKs is an autocatalytic reaction that occurs during protein synthesis and that generates a constitutively active kinase [32]. As DYRK activation does not depend on upstream kinases, other regulatory mechanisms are thought to operate. These include: the dephosphorylation of residues in the activation loop, although no phosphatase has been attributed this role to date, allosteric phosphorylation performed by other kinases [9,33,34,35,36], interactions with scaffolding proteins [37,38,39] or accessibility to substrates due to changes in the subcellular localization. In this regard, and given the constitutive nature of DYRK kinase activity, the regulation of their intracellular levels becomes crucial to modulate their functions, and thus, altering the DYRK expression acquires additional importance in terms of their impact on normal cell fitness.

## 3. The Role of DYRKS in Cancer

**Figure 2:**
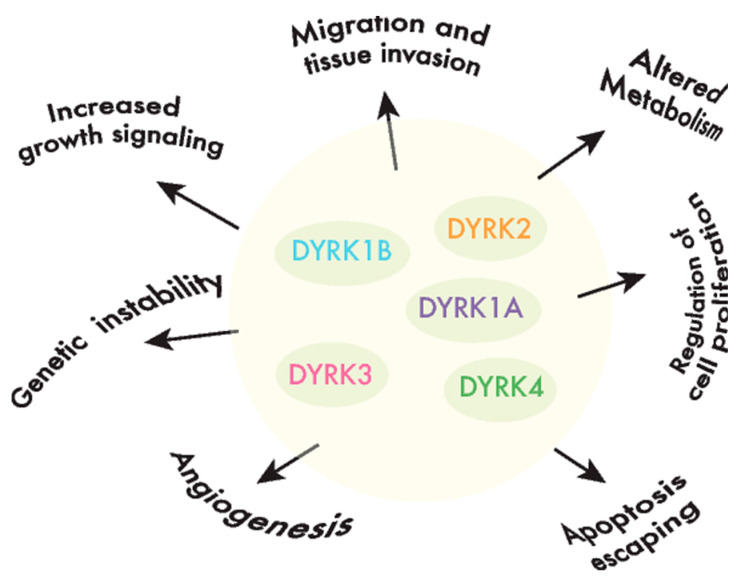
DYRKs are involved in cancer-associated processes. DYRK kinases participate in the regulation of crucial cell events, the perturbation of which is responsible for producing important features in cancer cells or the hallmarks of cancer.

DYRKs phosphorylate a wide range of substrates, including factors associated with one or several of the hallmarks of cancer [40] (Figure 2). Of all the DYRKs, only DYRK1A has been identified in high-throughput cancer studies, initially as a potential tumor suppressor using Tumor Suppressor and Oncogene Explorer (TUSON), a method developed to predict the potential of a given gene to act as a tumor suppressor, or oncogene, by computing somatic mutation profiles and copy number alterations (CNAs) [41]. Subsequently, it was proposed as a driver in liver cancer through a study that identified such drivers according to mutations in unusual nucleotide contexts [42]. Although these results would suggest that DYRKs are not major drivers of cancer, further evidence that they play a role in oncogenic processes has emerged over the past two decades. In the following sections, we will discuss the evidence indicating that each member of the DYRK family is involved in cancer by considering two main aspects: (i) alterations to the DYRK expression in tumor tissues, either based on published reports or on our own analysis of The Cancer Genome Atlas (TCGA: see Table S1; only cancer type cohorts with at least 10 paired samples, matched tumor-healthy tissue, were considered in the analysis), and (ii) the impact of DYRK-dependent phosphorylation on substrates involved in cancer-related events.

## 4. DYRK1A

The *DYRK1A* gene maps to chromosome 21, and it is the most extensively studied member of the family, mainly due to its key role in neurogenesis and in the etiology of some of the pathological traits associated to Down syndrome (DS: recently reviewed in [43]). In fact, *DYRK1A* is a dosage-sensitive gene, since small variations in the amount of its protein produce clinical phenotypes. On the one hand, DYRK1A is overexpressed 1.5-fold in DS individuals [22], and indeed, some of the morphological and cognitive defects of DS are reproduced when it is overexpressed in mouse models [43]. On the other hand, DYRK1A haploinsufficiency caused by de novo truncation or by missense-inactivating mutations was recently seen to underlie a rare, severe disorder, the DYRK1A haploinsufficiency syndrome (also known as MRD7 or Mental Retardation, Autosomal Dominant 7: OMIM#614104 and ORPHA:464311 and 268261; [44,45] and references therein).

**Figure 3:**
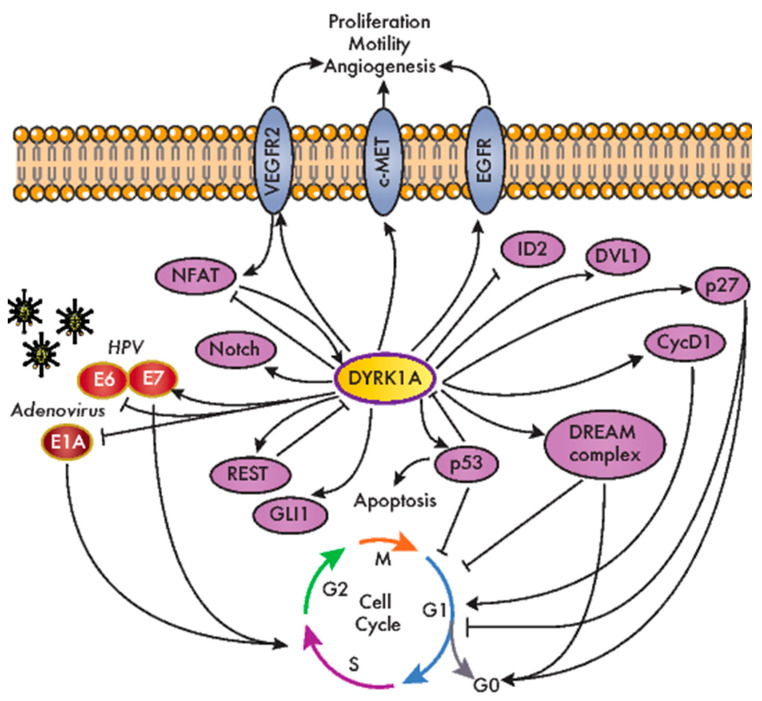
DYRK1A modulates the cellular factors involved in oncogenic processes. An overview of the DYRK1A interactions with the cellular factors involved neoplastic transformation and cancer-related pathways. CycD1: cyclin D1; DVL1: dishevelled 1; DREAM: dimerization partner (DP), RB-like, E2F and multi-vulval class B (MuvB); EGFR: epidermal growth factor receptor; HPV: human papilloma virus; ID2: inhibitor of DNA binding 2; NFAT: nuclear factor of activated T-cells; REST: RE1 silencing transcription factor; VEGFR2: vascular endothelial growth factor receptor 2.

DYRK1A is a pleiotropic factor that phosphorylates a broad set of proteins involved in many different cellular processes. These include factors that have been associated with all the hallmarks of cancer, from genomic instability to increased proliferation and resistance to programmed cell death or signaling pathways whose dysfunction is relevant to tumor onset and progression (e.g., Wnt, Notch and Hedgehog (Hh); Figure 3 and Table 1). Notably, the role of DYRK1A in specific cell responses has contrasting outputs, suggesting that it can act as a bimodal signaling regulator. For instance, DYRK1A stimulates the transcriptional activity of the Hh-signaling effector GLI1 through direct phosphorylation (Figure 3), although it also represses the Hh pathway through an indirect mechanism involving regulators of the actin cytoskeleton [46,47]. Likewise, DYRK1A negatively regulates the nuclear factor of activated T-cell (NFAT) transcription factors by inducing their phosphorylation-dependent nuclear export [48], yet it serves as a positive modulator of NFAT signaling in primary endothelial cells stimulated by vascular endothelial growth factor (VEGF) [49] (Figure 3). DYRK1A bimodal activity has also been reported in Wnt signaling, where DYRK1A acts as a positive regulator of the activated pathway, but it represses basal Wnt-signaling activity [21]. Finally, DYRK1A may induce cells to either enter or exit the cell cycle by controlling the Cyclin D1-to-p21 ratio [50]. All these observations may reflect the different experimental systems used by different groups, and specifically, the ectopic expression might produce confounding effects, since dramatic changes in the DYRK1A protein might be transiently induced in these cells over and above the endogenous levels. Alternatively, these findings might actually support the bimodal activity of DYRK1A in vivo, with the different outcomes depending on specific conditions such as cell identity, subcellular localization or the levels of kinase expression. Along similar lines, several studies have ascribed opposite functions to DYRK1A in cancer, reflecting a very complex scenario. Therefore, as will become evident below, it remains unclear as to whether DYRK1A acts as a tumor suppressor or a tumor promoter or, more probably, as either, depending on the tumor context.

### 4.1. DYRK1A and Cell Cycle Regulation

The first indications of a role for DYRK1A in cell immortalization were obtained in studies on oncogenic viruses, indicating that DYRK1A potentially affects cell transformation in oncovirus-associated cancer models. Both DYRK1A and DYRK1B interact with the adenovirus oncoprotein E1A, a feature conserved in the *Saccharomyces cerevisiae* DYRK Yak1p [71,72] (Figure 3). Mutations in E1A that interfere with DYRK1A binding produce hyper-transformation in conjunction with G12V HRAS proto-oncogene [72]. Moreover, the interaction between DYRK1A and E1A is dependent on the DCAF7 scaffold protein, which favors E1A phosphorylation at S89 [39] and contributes to the ability of the adenovirus to regulate the interferon response [73]. DYRK1A also interacts functionally with human papilloma virus (HPV), and *Dyrk1a* mRNA levels increase when primary mouse keratinocytes are immortalized by HPV infection (HPV high risk strain 16) [74]. Indeed, there is more DYRK1A protein in cervical lesions from HPV-derived patient samples than in the respective normal tissues. Alterations to the DYRK1A expression might involve the miR-1246 known to target DYRK1A [26], which is significantly downregulated in lesions from cervical cancer patients in a manner associated with HPV infection [75]. DYRK1A interacts and phosphorylates HPV16 E7, stabilizing E7 and thereby potentially promoting E7-dependent cell proliferation [76] (Figure 3). Moreover, DYRK1A interacts with beta-HPV E6 proteins (Figure 3), and this DYRK1A interaction is defective in HPV E6 variants found in invasive cervical carcinoma [77].

The link between DYRK1A and cell proliferation is based on its ability to phosphorylate crucial cell cycle regulators, like Cyclin D proteins or p27 (Figure 3 and Table 1), modulating their stability and, hence, their cellular levels [50,53,58,78]. It should be noted that these regulatory mechanisms have mainly been observed in nontransformed cells, and as mentioned above, the effect of DYRK1A on the cell cycle is not straightforward, as it depends on the Cyclin D-induced stabilization of the CDK inhibitor p21, at least for Cyclin D1 [50]. In addition, DYRK1A is a kinase in the DREAM complex (dimerization partner (DP), RB-like, E2F and multi-vulval class B (MuvB)). DYRK1A promotes the assembly of this complex by phosphorylating the DREAM component Lin52 on S28, thereby triggering cell cycle exit [54] (Figure 3). Notably, DYRK1A-mediated DREAM complex formation was proposed to be responsible for ovarian cancer cell dormancy [79] and for the quiescence of gastrointestinal stromal tumor (GIST) cells induced by treatment with imatinib [80]. Another DYRK1A cell cycle-related target is the p53 tumor suppressor (Figure 3). DYRK1A positively regulates p53 transcriptional activity by the direct phosphorylation of S15 [60], but it also negatively regulates this factor by enhancing sirtuin (Sirt)1-dependent deacetylation [81]. The functional interaction of DYRK1A with p53 promotes cell cycle arrest in embryonic neuronal cells [60], as well as the survival of osteosarcoma and colorectal cancer (CRC) cell lines in response to genotoxic stress [81]. The cross-talk between DYRK1A and p53 also involves a negative feedback loop that engages two distinct regulatory mechanisms: (i) the p53-dependent induction of miR-1246, which suppresses DYRK1A expression [26], and (ii) the degradation of the DYRK1A protein mediated by the E3 ubiquitin ligase mouse double-minute 2 homolog (MDM2) [82].

### 4.2. DYRK1A and Receptor Tyrosine Kinase (RTK)-Dependent Signaling

An important aspect of the participation of DYRK1A in oncogenic processes is related to the regulation of RTK-dependent signaling. This class of protein kinases is frequently altered in tumors, with almost half of them included in the list of driver kinases assembled in 2016 [2]. We found a conserved regulatory pattern that involves the positive effects of DYRK1A on the stability of several RTKs (Figure 3 and Table 1), yet it is unclear whether these effects are mediated by a shared DYRK1A target or one specific to each RTK. Thus, DYRK1A prevents epidermal growth factor receptor (EGFR) endocytosis-mediated degradation in neural stem cells [65] and indeed, DYRK1A-dependent EGFR stabilization has been described in glioblastoma (GBM) and non–small cell lung cancer (NSCLC) cell lines [66,67]. Indeed, DYRK1A and EGFR protein levels correlate in tissues from glioma patients [66]. In pancreatic ductal adenocarcinoma (PDAC) tumor tissue, a similar relationship was found between the expression of DYRK1A and c-MET, the hepatocyte growth factor receptor [68]. DYRK1A exerts a positive role on the c-MET protein levels in cell models of PDAC and NSCLC, which might contribute to the protumorigenic role of DYRK1A in these types of tumors [67,68]. Given that RTKs are common targets in cancer therapy [83], the inhibition of DYRK1A (and its paralog DYRK1B) could be considered an element in combinatorial therapies to simultaneously target several deregulated RTKs. Finally, DYRK1A depletion reduces the levels of membrane-bound VEGF receptor 2 (VEGFR2), and it causes defects in VEGFR2-dependent signaling and the downstream NFAT-dependent transcriptional response in endothelial cells [49]. These results are correlated with the defects in developmental angiogenesis in a mouse model in which the *Dyrk1a* dosage is reduced [49], although whether DYRK1A has a proangiogenic role in the tumor microenvironment needs to be further explored.

DYRK1A regulates other cell factors known to participate in malignant transformations, including the stemness-related RE1 silencing transcription factor REST [84] or key effectors of cancer-promoting signaling pathways, like the Hh, Wnt and Notch pathways (Table 1). However, whether DYRK1A is connected to alterations in these pathways during tumor initiation/progression has not yet been established.

### 4.3. DYRK1A in Cancer

Changes in the *DYRK1A* expression have been analyzed in tumor samples, and as such, *DYRK1A* was seen to be downregulated in breast cancer [27] and in acute myeloid leukemia (AML) tissue [55], and it is upregulated in GBM [66], lung cancer [67] and head and neck squamous cell carcinoma (HNSCC) [85], as well as in PDAC [68]. Indeed, a weaker DYRK1A expression was correlated with a worse overall survival in breast cancer patients [27] and a poorer prognosis in CRC and GBM patients [62,86], whereas more DYRK1A was associated with a reduced survival time in patients with lung cancer [67]. Our analysis of the TCGA RNA-Seq data revealed a clear trend towards *DYRK1A* downregulation in tumor tissues, with a significant downregulation of *DYRK1A* in 11 out of the 15 tumor types considered (Table S1): colon (COADREAD), esophagus (ESCA), HNSCC, kidney (KIRP and KIRC), liver (LIHC), lung (LUSC and LUAD), stomach (STAD), thyroid (THCA) and uterus (UCEC). No significant CNAs associated with changes in the gene expression were observed (Table S1), suggesting that the changes in RNA levels could be due to epigenetic, transcriptional or post-transcriptional alterations. The general trend towards a reduced *DYRK1A* expression in tumor samples would be in agreement with a more prominent tumor-suppressor role, even though the correlation between *DYRK1A* mRNA and the protein levels has not been properly evaluated in any cancer study.

A direct role for DYRK1A in tumor progression has been proposed in several studies. In cell models, DYRK1A knockdown or enzymatic inhibition reduced the proliferation of HNSCC cell lines [85], luminal/HER2 breast cancer [87] or PDAC [68], as well as impaired the self-renewal capacity of GBM cells [66] and compromised ovarian cancer spheroid cell viability [79]. The pro-oncogenic role suggested by these findings is in accordance with results obtained from xenografts in mouse models [66,68,85]. However, a tumor suppressor role was also proposed on the basis of DYRK1A overexpression experiments in AML cells [55]. The antitumor role of DYRK1A was suggested to be related to the lower incidence of cancer in DS individuals [88], deviating from that observed in the normal population. Indeed, epidemiological studies have demonstrated that individuals with DS have a markedly lower incidence of most solid tumors [89] and reduced cancer-associated mortality [90] relative to the age-adjusted non-DS population. However, childhood leukemia represents a strong exception to this trend, as DS children have a 10 to 50-fold increased risk of developing AML, as well as a 500-fold increased incidence of developing acute megakaryoblastic leukemia (AMKL) [91]. In this regard, DYRK1A was proposed to be a potent, megakaryoblastic oncogene, suggesting that NFAT-negative regulation through an imbalance in DYRK1A might perturb myeloid differentiation and promote AMKL in DS individuals [92].

In summary, the literature reflects a complex picture in which DYRK1A may fulfill opposite roles in different tumor contexts. Thus, more research is clearly required to fully understand how DYRK1A contributes to tumor initiation or progression.

## 5. DYRK1B

**Figure 4:**
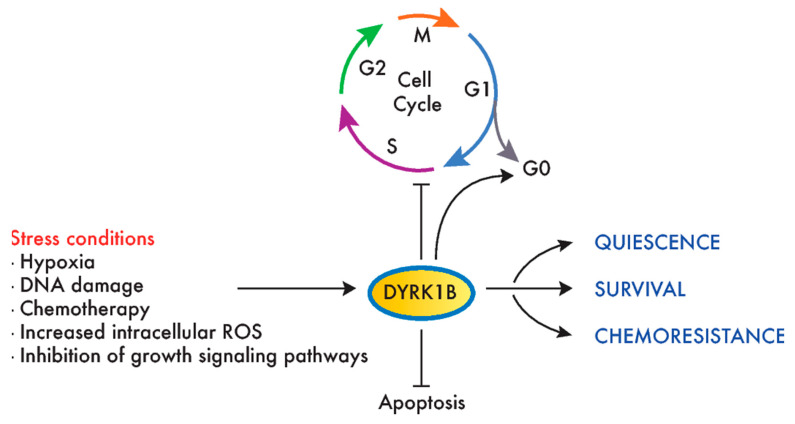
DYRK1B promotes survival and chemoresistance in cancer cells. Environmental stress conditions induce DYRK1B expression or activity in tumor cells, which, in turn, promotes cell cycle exit, quiescence (entry in G0) and survival. This mechanism has been proposed to mediate the resistance to chemotherapeutic agents that target dividing cells.

DYRK1B is the closest paralog to DYRK1A, sharing 85% homology that extends beyond the kinase domain (Figure 1A). Although both kinases share substrates (Table 1), the distinct clinical outcome of inactivating mutations indicates they are not functionally redundant, i.e., a disorder within the autism spectrum for DYRK1A and a metabolic syndrome for DYRK1B (abdominal obesity metabolic syndrome-3, OMIM#615812; [93]). A recent review of DYRK1B has offered extensive information on this kinase [94], and thus, here, we will focus on those aspects of the kinase that are related to its role in cancer, which, unlike DYRK1A, point mostly to a prosurvival and protumorigenic role for DYRK1B (Figure 4).

The first studies into the influence of DYRK1B in cancer suggested a role in the survival of cancer cells, with a stronger DYRK1B expression in CRC samples than in normal tissue [95]. Several studies extended this finding to other tumor types, included liposarcoma [96], rhabdomyosarcoma [97], osteosarcoma [98], lung [99], breast [100], ovary [101] and PDAC [68,102,103]. Indeed, a differential expression analysis using TCGA data finds *DYRK1B* to be overexpressed in several tumor types, including bladder (BLCA); breast (BRCA); kidney (KICH, KIRC and KIRP); liver (LIHC); prostate (PRAD); thyroid (THCA) and uterus (UCEC) (Table S1). Furthermore, we confirmed previous reports on the amplification of the *DYRK1B* genomic region (19q13.2) in ovarian cancer [104,105] and PDAC [102,106] (Table S1). The amplification of this region with coherent DYRK1B overexpression was observed in other tumor types (Table S1), suggesting that they may underlie the increase in *DYRK1B* expression, although this may also be provoked by transcriptional activation due to changes in the transcriptional profiles of tumor cells [107,108,109,110,111].

The functional interaction of DYRK1B with signaling pathways involved in cancer cell proliferation has been explored, assessing both the fluctuations in DYRK1B expression upon the perturbation of growth pathways and the output provoked by DYRK1B depletion in cancer cell lines. Several findings point to an antagonistic role of DYRK1B and MAPK signaling, with an increase in DYRK1B in response to inhibitors of the MAPK kinase (MEK) in CRC and melanoma cell lines [36,95] and a reduction following the mitogen activation of the RAS-MEK-extracellular signal-regulated kinase (ERK) pathway in skeletal myoblasts [112]. The cross-talk between DYRK1B and the MAPK pathway was further explored in ovarian cancer and NSCLC cell lines, where DYRK1B knockdown increased c-RAF and ERK activation [107]. This DYRK1B-MAPK cross-talk might be even more complex, since DYRK1B is an ERK substrate at a residue that potentiates DYRK1B activity [36], and accordingly, oncogenic KRAS mutants act as positive modulators of DYRK1B activity [113,114]. The RAS-DYRK1B axis was proposed to participate in both autocrine and paracrine Hh signaling in PDAC [114], although the role of DYRK1B in the regulation of Hh signaling in cancer remains controversial, as it has been attributed opposite functions within this signaling pathway [61,114,115]. Finally, there also appears to be cross-talk between DYRK1B and the mammalian target of rapamycin (mTOR) pathway, with DYRK1B expression upregulated upon mTOR inhibition [109] and mTOR/AKT activation induced by DYRK1B within the Hh signaling pathway in pancreatic and ovarian cancer cells [115].

Like DYRK1A, DYRK1B phosphorylates several cell cycle regulators, like Cyclin D1, p21, p27 and Lin52 [52,54,57,59] (Table 1). In this context, DYRK1B overexpression may help maintain a reversible quiescent state or inhibit cancer cell proliferation [116,117,118], while DYRK1B reduction can drive cell cycle entry in quiescence (by reducing the DYRK1B expression in PDAC or by DYRK1B inhibition in CRC cell lines) [119]. By contrast, the depletion of DYRK1B in HPV E7-expressing keratinocytes interferes with the induction of the S-phase promoted by E7 [120]. In addition, the depletion or inhibition of DYRK1B enhances the DNA damage, apoptosis and sensitivity to reactive oxygen species (ROS) or chemotherapeutic drugs targeting proliferating cells [15,104,121,122,123], as well as the sensitivity to compounds that target pathways favoring proliferation in cell lines of different tumor origins, such as mTOR and MEK inhibitors [107,109] (Figure 2).

A protumorigenic role for DYRK1B has been proven in cellular models of ovarian and pancreatic cancers. Thus, DYRK1B knockdown negatively affects different aspects of ovarian cancer cell malignancy, including viability, proliferative potential and migratory capacity [1,124,125]. Likewise, DYRK1B knockdown negatively affects PDAC cell proliferation, migration and invasion [68,102], whereas a treatment of PANC1 xenografts with a DYRK1B inhibitor impairs tumor growth [103]. In summary, and in contrast to the controversial role of DYRK1A in cancer, clear oncogenic facets have been attributed to DYRK1B, acting as a prosurvival factor that could help cancer cells survive in suboptimal growth conditions and preventing chemotherapeutic-induced DNA damage and apoptosis.

## 6. DYRK2

DYRK2 is a class II DYRK that has been more intensely studied in terms of its involvement in the events associated with tumor progression. The biochemistry and biology of DYRK2 was covered in recent reviews [126,127], and thus, here, we will center on the activity of this kinase in the context of tumor biology.

### 6.1. Altered DYRK2 Expression in Cancer

The first hints that DYRK2 may influence carcinogenesis were derived from a genomic analysis and differential gene expression studies, highlighting *DYRK2* overexpression in association with the amplification of its genomic locus in esophageal and lung adenocarcinomas [128,129], GIST [130], gastric adenocarcinoma [131] and liposarcoma [132]. Additional evidence for the involvement of DYRK2 in cancers came from a germline-somatic association study of genetic alterations in multiple cohorts of breast cancer patients [133]. Moreover, the upregulation of DYRK2 was described in triple-negative breast cancer (TNBC) and multiple myeloma [134]. Conversely, DYRK2 was downregulated in lung adenocarcinoma and squamous cell carcinoma [135], diffuse large B-cell lymphoma [136], CRC [137], hepatocellular carcinoma (HCC) [138,139] and high-grade glioma [140]. Our analysis of TCGA data found *DYRK2* to be overexpressed in eight tumor cohorts: bladder (BLCA), breast (BRCA), esophagus (ESCA), kidney (KIRC and KIRP), liver (LIHC), lung (LUAD and LUSC) and stomach (STAD) (Table S1). An analysis of CNAs indicated that *DYRK2* upregulation might be associated with gene amplification in BLCA, BRCA, LUAD, LUSC and STAD (Table S1). Moreover, a significant *DYRK2* overexpression in *DYRK2*-amplified tumors was also observed in HNSCC, ovary (OV), melanoma (SKCM) and sarcoma (SARC) (Table S1). Notably, no correlation was observed between the DYRK2 protein and mRNA levels in breast cancer tissue when compared with healthy tissue [141]. A similar discrepancy between the DYRK2 protein and mRNA was also detected in liver and lung cancers between a published protein data analysis and our TCGA analysis of mRNA changes (Table S1). Hence, post-transcriptional mechanisms may play a crucial role in determining the levels of the DYRK2 protein in tumor cells, and these might explain, at least in part, the conflicting data obtained in relation to breast cancer (see below).

Besides the alterations to the DYRK2 expression, it has been proposed that this kinase may represent a prognostic marker for different types of cancer, based on a correlation analysis between the gene/protein expressions and distinct clinical features like the degree of malignancy, relapse, response to chemotherapy or patient survival. Thus, higher DYRK2 levels were positively correlated with a more favorable prognosis and better response to chemotherapy in lung and bladder cancer patients [142,143,144] and with better survival in patients with CRC liver metastases [145]. Likewise, a weaker DYRK2 expression was associated with a worse prognosis in ovarian serous adenocarcinoma [146], CRC [137,145], HCC [138,139], glioma [140] and non-Hodgkin’s lymphoma [136] patients. The situation in breast cancer is less clear, with conflicting results. As mentioned above, such discordance may be due to the use of mRNA or protein to assess the DYRK2 expression. As such, DYRK2 protein levels were shown to inversely correlate with tumor invasiveness [56], and enhanced 10-year disease-free survival was evident in DYRK2-positive breast cancer patients when compared to DYRK2-negative patients [147], while a stronger DYRK2 mRNA expression was associated with a worse prognosis in another study of breast cancer patients [148]. Apart from this, and in general, it appears that the weaker the expression of DYRK2, the worse the prognosis. This model is consistent with experimental data when DYRK2 levels are manipulated in carcinoma cell lines (ovary, CRC and HCC) that are then used as xenografts in mice, whereby DYRK2 gene silencing confers an enhanced proliferative capacity and metastatic potential in vivo [139,145,146]. However, again, some discordant phenotypes have been described in vivo when studying DYRK2-depleted breast cancer cell lines.

A few works have explored the mechanisms underlying the reduction of DYRK2 levels in tumor cells. The Kruppel-like factor 4 transcription factor has been shown to repress DYRK2 expression, acting directly on the DYRK2 promoter in chronic myeloid leukemia (CML) cell lines and mouse models, thereby favoring tumor progression [149]. Moreover, the DNA-methyltransferase 1-dependent methylation of the *DYRK2* promoter provokes transcriptional downregulation that may influence DYRK2 expression in CRC cells [150]. DYRK2 protein levels are also modulated by several E3 ubiquitin ligases, including seven in absentia homolog 2 (SIAH2) and MDM2 [9,151]. Interestingly, the DDR protein kinase ATM is involved in this process by phosphorylating DYRK2 and, thus, preventing DYRK2 degradation mediated by MDM2 [9,151]. This relationship could contribute to the ability of these E3 ubiquitin ligases to promote survival in states of hypoxia and in the face of DNA-damaged stress, respectively, by suppressing the proapoptotic activities of DYRK2. In particular, mutual regulation has been described for SIAH2 and DYRK2 [151]; indeed, an increase in the SIAH2 protein has been observed in lung cancer tissue and linked to DYRK2 downregulation [135]. Besides the alterations to the cellular levels of DYRK2, changes in the substrate selectivity have been seen in relation to Snail in ovarian cancer, with DYRK2 phosphorylation prevented by the prior p38-mediated phosphorylation of Snail [152].

### 6.2. The Molecular Mechanisms Underlying the Role of DYRK2 in Cancer Cells

**Figure 5:**
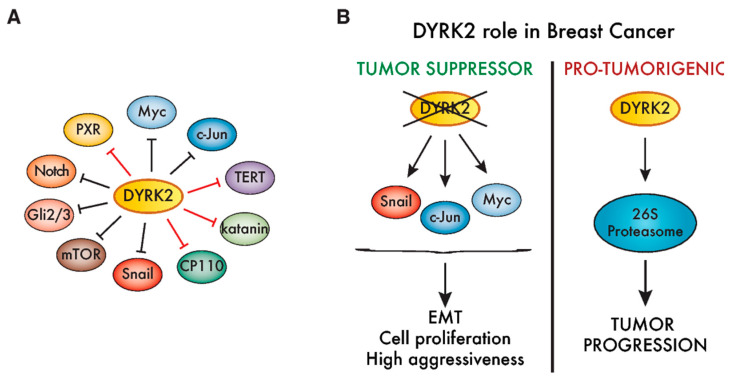
Cancer-associated activities of DYRK2. (**A**) DYRK2 substrates are associated with different aspects of tumorigenesis, including proliferation (Myc, c-Jun, centrosomal protein of 110 kDa (CP110) and katanin: [38,56]); transformation (TERT: [154]); invasiveness (Snail: [141]); signaling (mTOR, Notch and Gli2/3: [34,64,155]) or the xenobiotic response system (pregnane X receptor (PXR): [156]). Red lines mark those substrates that are degraded when DYRK2 associates with the multicomponent E3 ubiquitin ligase EDVP. The rest (black lines) are all targets of FBXW7, considered to be a tumor suppressor [157]. (**B**) DYRK2 has been proposed as both a protumorigenic factor, as well as a tumor suppressor, in breast cancer. On the one hand, reduced levels of DYRK2 enhance the accumulation of mitogenic transcription factors like c-Jun and Myc, as well as the epithelial-to-mesenchymal transition (EMT)-promoting factor Snail, which is correlated with tumor progression and more aggressiveness. On the other hand, DYRK2 phosphorylates and positively regulates the 26S proteasome, promoting triple-negative breast cancer (TNBC) cell growth, and thus, DYRK2 depletion or inhibition impairs tumor growth in vivo.

Several clues have been obtained regarding the putative molecular mechanisms responsible for DYRK2-mediated tumor development/progression. Thus, DYRK2 activity appears to affect crucial processes like the cell cycle, DDR, epithelial-to-mesenchymal transition (EMT), the xenobiotic response system and cellular proteostasis [127]. The activity of DYRK2 has often been linked to its ability to negatively regulate the stability of its target proteins—in particular, through its interaction with the UBR5/EDD-DNA damage-binding protein 1 (DDB1)-DDB1- and cullin 4-associated factor homolog 1 (DCAF1/VPRBP) (EDVP) E3 ubiquitin ligase complex [38] (Figure 5A). The relevance of this interaction is highlighted by the alterations in the assembly of the EDVP complex detected in the analysis of certain DYRK2 mutants found in cancer samples [153]. In addition, it is worth noting that many of the proteins that are degraded following DYRK2 phosphorylation are targets of the tumor suppressor F-box/WD repeat-containing protein 7 (FBXW7) (Figure 5A), suggesting possible cross-talk with E3 ubiquitin ligase protein complexes made up of this factor. As previously mentioned, DYRK2 and SIAH2 cellular levels inversely correlate [151], further supporting a regulatory cross-talk between DYRK2 and several E3 ubiquitin ligases. Moreover, phosphorylation of the 19S subunit PMSC4/Rpt3 [148] might also contribute to the DYRK2-dependent modulation of protein accumulation.

In conjunction with the reduced expression of DYRK2 in tumor samples, DYRK2 depletion promotes the proliferation of cell lines originating from distinct tumor types, including breast, lymphoma, osteosarcoma, CRC and HCC [56,136,137,138,139,145,158], suggesting that DYRK2 may acts as a brake on proliferation. In this regard, DYRK2 phosphorylates the oncogenic pro-proliferative transcription factors c-Jun and Myc, increasing their rate of degradation [56]. Indeed, DYRK2 levels negatively correlated with c-Jun/Myc levels in breast tumor tissues [56] (Figure 5B). Other DYRK2 targets associated with cell cycle regulation are the centrosomal proteins katanin p60 and CP110/CCP110 and the telomerase TERT (Figure 5A), although no specific link between these proteins and DYRK2-dependent tumorigenic processes has as yet been proposed [38,154,159].

Besides the cell cycle, DYRK2 also regulates cell factors involved in other processes crucial for tumor progression, such as apoptosis or DDR. The interaction between DYRK2 and the E3 ubiquitin ligase RNF8 was proposed to influence DYRK2 recruitment to the DNA repair machinery [160], and the phosphorylation of p53 by DYRK2 promotes apoptosis in response to DNA damage, with ATM acting upstream by increasing the DYRK2 nuclear accumulation [9,19]. The modulation of p53 and Myc was also proposed as a DYRK2-mediated mechanism in leukemia stem cells and CML cell lines [149]. The putative regulatory activity during DDR and/or the ability of DYRK2 to increase components of the xenobiotic response system, such as the PXR/NR1I2 nuclear receptor [156] (Figure 5A), may contribute to enhance the resistance to chemotherapy drugs observed upon DYRK2 silencing [64,138,146]. A reduction in DYRK2 has also been linked to the enhanced migration and invasion of breast, glioma and ovary cancer cell lines [56,64,140,141,146]. In this regard, DYRK2 phosphorylates the EMT transcription factor Snail, priming it for ubiquitination-mediated degradation [141] (Figure 5A), which provides additional evidence that DYRK2 prevents the activation of aggressive phenotypes in breast and ovarian cancer cells.

To date, the most controversial role for DYRK2 associated with tumors is in breast cancer (Figure 5B). Based on results from xenograft experiments using MCF-7 cells, a tumor-suppressor role was first proposed given that DYRK2 silencing favored tumor growth [56]. The enhanced expression of direct DYRK2 targets like c-Jun or Myc, and/or other proteins like CDK14, could account for this phenotype [56,158]. Similarly, DYRK2 silencing increased the invasion, metastasis [141] and breast cancer cell stemness [161]. Conversely, using a clustered regularly interspaced short palindromic repeats (CRISPR)-based approach to generate DYRK2-knock out MDA-MB-468 breast cancer cells, DYRK2 was seen to promote breast cancer cell proliferation and tumor growth in xenografts. This effect could be mediated by the DYRK2-dependent phosphorylation of the proteasomal 19S subunit PMSC4/Rpt3 [148] (Figure 5B). In this context, two DYRK2 inhibitors, the natural drug curcumin and the small-molecule LDN192960, impaired cell proliferation and invasion and induced apoptosis in multiple myeloma and TNBC cell lines [134,162]. Whether these contradictory results arise from the use of cell lines with different responsiveness to estrogen/progesterone and/or an “addiction” to proteasome activity must be further explored. In any case, the DYRK2-associated stratification of breast tumors should be properly studied before designing any DYRK2-targeting therapeutic approach.

## 7. DYRK3 and DYRK4

The contribution of DYRK3 and DYRK4 to tumorigenesis is less clear, with very little evidence for the participation of DYRK3 and almost no evidence for that of DYRK4. This lack of information also mirrors the limited knowledge of the biological activities of these two family members.

DYRK3 was initially described as a kinase involved in erythroid development [24,163], although its most relevant activity described to date is the ability to regulate phase-transition during mitosis, thereby mediating the formation of multiple liquid-unmixed compartments such as stress granules, an essential process for proper mitotic division [16,17]. The association of DYRK3 with the mTORC1 pathway was established through the ability of DYRK3 to phosphorylate PRAS40, thereby promoting mTORC1 activity [16].

Our analysis of the TCGA data did not reveal any specific trend for DYRK3, which is under-expressed in breast (BRCA), kidney (KIHC), lung (LUAD and LUSC), prostate (PRAD) and thyroid (THCA) tumor cohorts and overexpressed in colon (COADREAD), HNSCC, kidney (KIRC) and stomach (STAD) cancer tissues (Table S1). Likewise, no particular trend can be found in the literature. Thus, *DYRK3* mRNA was found significantly increased in highly invasive NSCLC cell lines compared with low invasive lines [164], while a strong DYRK3 expression was positively correlated with survival in glioma patients [165]. Moreover, DYRK3 was proposed as a specific early-stage tumor driver in gastric cancer [166]. Finally, a reduction in the DYRK3 protein was recently described in HCC biopsies relative to normal tissue, and low DYRK3 levels were associated with a poor prognosis in this type of cancer [167]. In addition, manipulating the DYRK3 expression in HCC cells demonstrated an inverse correlation with proliferation rates both in vitro and in tumor xenograft models, as well as with the metastatic potential of the tumor cells, further evidencing that DYRK3 fulfills a tumor-suppressor role in this type of cancer [167]. Indeed, a regulatory axis was proposed that involves the ATF4 transcription factor and its coactivator NCOA3 as a direct DYRK3 substrate, regulating the expression of key metabolic enzymes in the purine synthesis pathway that are relevant to HCC progression. However, whether this role for DYRK3 can be extrapolated to other tumors remains to be confirmed.

DYRK4 is the DYRK family member associated with the least significant alterations in the TCGA cohorts analyzed. We found that it was downregulated in lung (LUAD), prostate (PRAD) and stomach (STAD) cohorts and with different patterns of expression in the three kidney cohorts: overexpressed in KIRC and KIRP and downregulated in KIHC (Table S1). Interestingly, a recent high-throughput screen on 313 kinase-deficient cell lines revealed that DYRK4 knockout cells were among the most sensitive to agents that produce DNA damage [168], suggesting that DYRK4 might merit further exploration as a putative target to enhance chemotherapy toxicity on cancer cells.

## 8. DYRK Inhibitors as Antitumor Therapies

Chemical compounds that bind and functionally block protein kinases have been studied extensively and employed as antitumor agents, both in research and in clinical trials [83]. Although the role of DYRK family members in tumorigenesis and tumor progression has not been fully elucidated, pharmacological inhibitors of DYRK kinases have been tested in laboratories for their antimalignant activity, and a few of them are already undergoing clinical trials.

In the case of DYRK1A, the search for both naturally occurring and synthetic inhibitors has been extensive given that DYRK1A may be a potential pharmacological target not only in cancer but, also, in neurodegenerative diseases (reviewed in [43]), DS [169,170,171,172] and diabetes (reviewed in [173]). DYRK1A inhibitors have been comprehensively reviewed elsewhere [174,175,176], and so, we will only refer to the orally bioavailable archetypic DYRK1A inhibitors in tumor contexts. For instance, the anticancer properties of green tea and its derivatives have been proven in many animal models, a product that contains the natural DYRK1A inhibitor Epigallocatechin-3-gallate (EGCG). However, EGCG can potentially target many different intracellular pathways [177], making it difficult to assign particular effects to DYRK1A inhibition. Additionally, the ß-carboline alkaloid harmine selectively inhibits DYRK1A and—albeit, less efficiently—other members of the family [178,179], and it has been reported to have cytotoxic effects on cancer cell lines [66,85,180,181] and antitumor effects in vivo in glioma and in PDAC xenograft experiments [66,68], as well as synergistic effects with other chemotherapeutic agents [79,80,182]. However, the neurotoxic effects of harmine due to the targeting of monoamine oxidase A rule against its use in humans. Therefore, the search for harmine derivatives with enhanced antitumor activity and reduced neurotoxic effects has been intense in recent years [183,184,185]. Finally, the synthetic DYRK1A inhibitor INDY, proven to modulate the phenotypic effects of DYRK1A overexpression in vivo [186], has been shown to improve the response of ovarian cancer spheroids to carboplatin [79].

Compounds targeting DYRK1B, with either restricted or broad specificity, have been used as research tools, and they display toxicity towards several types of cancer cells or they promote the cell cycle re-entry of quiescent tumor cells (reviewed in [94]). The latter would enhance the effectiveness of other antiproliferative drugs in combinatorial approaches. For instance, the DYRK1B inhibitor AZ191 [52] increases the anticancer effects of doxorubicin in liposarcoma cell lines [96] or sensitizes the PDAC cell lines to mTOR inhibition [115]. However, AZ191 has been also shown to counteract the antitumor effects of the lysosome inhibitor Bafilomycin A1 in HCC cell lines [111]. For DYRK2, experimental data on the antitumor effects of the natural DYRK2 inhibitor curcumin and of the synthetic compound LDN192960 was obtained in both in vitro and in vivo models of TNBC and multiple myeloma, supporting the hypothesis that DYRK2 is a promising pharmaceutical target in these malignancies [134,162]. Finally, better understanding the role of DYRKs in tumor cells has proven valuable by helping to identify combinatorial therapeutic approaches, as in the cases of the DYRK1B inhibitors that enhance the inhibitory efficiency of MEK and mTOR [107,109,187] or DYRK2 inhibition sensitizing MDA-MB-468 cells to the proteasome inhibitor bortezomib [148].

Most kinase inhibitors lack complete specificity [178,188], a potentially negative property that might be exploited in multitargeting strategies, which become a familiar situation in antitumor therapies. Interestingly, the only inhibitors of the DYRK family members currently being screened in clinical trials were identified as inhibitors of other protein kinases. In particular, compound CX-4945 was initially identified as a casein kinase 2 inhibitor, but it was subsequently shown to be a potent DYRK1A and DYRK1B inhibitor [171], and it is currently in phase I and II clinical studies for medulloblastoma, cholangiocarcinoma and basal cell carcinoma (NCT02128282, NCT03904862 and NCT03897036). Recently, OTS167, a chemical initially described as a maternal embryonic leucine zipper kinase inhibitor, has been proven to have potent anti-DYRK1A activity [189]. OTS167 is currently being assessed in clinical trials for the treatment of advanced breast cancer and TNBC (phase I) and for multiple types of leukemia, including AML and advanced CML (phase II: NCT02795520). Finally, two other DYRK inhibitors have been assessed in clinical trials for non-neoplastic disorders: GSK-626616 [16] completed a phase I clinical trial to evaluate its action on anemia (NCT00443170), and lorecivivint, a potent CLK2 inhibitor that also inhibits DYRK1A [190], is being studied in a phase II trial for the treatment of moderate-to-severe symptomatic osteoarthritis (NCT03706521). Thus, they could be repurposed in trials for the treatment of specific cancer types.

## 9. Conclusions

In the last decade, more experimental evidence indicates that DYRK protein kinases are a novel class of “kinase-of-interest” in cancer. However, this evidence mostly comes from studies exploring DYRK expressions in tumor tissues and/or the phenotypic changes triggered by manipulating the DYRK protein in cancer cell lines. These data not only provide a partial and confusing picture of the influence of DYRKs in tumor initiation and progression, but also, they highlight the many questions that still need to be addressed. In particular, it remains unclear which molecular pathways are regulated by DYRKs in different tumor types and which of them selectively trigger cells to engage in neoplastic transformation or enhance the malignant phenotype of tumor cells. Resolving these issues will not only help understand the biology behind the activity of these kinases, but also, it will provide a basis for the rational design of therapeutic approaches based on inhibitors. In this regard, while incomplete, the currently available data provides precious information on which forthcoming therapeutic approaches may be based. Therefore, the tumor types in which downregulation of the DYRK kinase has been associated with increased tumor growth and/or invasiveness should not be considered for trials with DYRK inhibitors. Conversely, inhibitors targeting DYRK family members that are known to favor the tumorigenesis of specific tumor types should be considered for such trials. Nevertheless, putative side effects due to the inhibition of members that are essential to maintaining cellular homeostasis in normal cells, such as the dosage-sensitive DYRK1A or DYRK1B, should be carefully monitored. In this context, engineering drugs to increase their specificity, exclusively targeting proliferating cells, would be worthwhile. Finally, and considering the differential and sometimes opposite roles of distinct DYRK kinases in tumor progression, selectivity towards a specific member of the family is crucial and, at the same time, very challenging, particularly given the strong structural similarity of the catalytic domain. Smart solutions might include an allosteric drug design or other additional efforts to increase compound selectivity.

To conclude, many important advances in understanding how the dysregulation of DYRK protein kinases is associated to pathological phenotypes in humans have been made in recent years—in particular, in terms of the involvement in DYRK cancers. Still, many secrets behind the oncogenic or protective potential of DYRK kinases remain to be revealed, and we anticipate that the field will continue to grow for the foreseeable future.

## Figures and Tables

**Table 1:** Signaling molecules targeted by DYRK1A and other dual-specificity tyrosine-regulated kinases (DYRKs).

Signaling Pathway	Target	Role	Reference	Other DYRKs	Role	Reference
Ca2+-NFAT	NFAT	Negative regulation of nuclear accumulation	[48]	DYRK2	same	[51]
Cell cycle	Cyclin D1	Negative regulation of protein levels	[50]	DYRK1B	same	[52]
	Cyclin D2	Negative regulation of protein levels	[53]	n.d.	n.d.	-
	Cyclin D3	Negative regulation of protein levels	[53]	n.d.	n.d.	-
	Lin52	Positive regulation of DREAM complex assembly	[54]	DYRK1B	same	[54]
	Myc	Negative regulation of protein levels	[55]	DYRK2	same	[56]
	p21	n.d.	-	DYRK1B	Negative regulation of protein accumulation	[57]
	p27	Positive regulation of protein levels	[58]	DYRK1B	same	[59]
	p53	Positive regulation of transcriptional activity	[60]	DYRK2	same	[19]
Hedgehog	ABLIM1	Negative regulation of F-actin formation	[47]	n.d.	n.d.	-
	GLI1	Positive regulation of nuclear accumulation and transcriptional activation	[46,47]	DYRK1B	Positive regulation of protein accumulation	[61]
	GLI2/3	n.d	-	DYRK2	Negative regulation of protein accumulation	[34]
Hypoxia	ID2	DYRK1A-mediated phosphorylation of ID2 leads to HIF2α destabilization	[62]	DYRK1B	same	[62]
	EGLN2/PHD1 *	Interaction enhances DYRK1A phosphorylation of ID2	[62]	DYRK1B	same	[62]
Notch	Notch1	Negative regulation of transcriptional activity	[63]	DYRK2	same	[64]
	Notch1	Negative regulation of protein levels	[64]	DYRK1B, DYRK2	same	[64]
RTKs	EGFR *	Positive regulation of protein levels	[65,66,67]	n.d.	n.d.	-
	c-MET *	Positive regulation of protein levels	[67,68]	DYRK1B	same	[68]
	VEGFR2 *	Positive regulation of protein levels	[49]	n.d.	n.d.	-
Wnt	β-catenin	Regulation of binding to co-activators p300 or CBP	[69]	n.d.	n.d.	-
	Catenin-p120	Positive regulation of Wnt signaling	[70]	n.d.	n.d.	-
	DKK3	n.d.	[21]	n.d.	n.d.	-
	DVL1	Positive regulation of DVL1-dependent induction of a TOP-FLASH reporter	[21]	n.d.	n.d.	-

* Not a direct phosphorylation target. ABLIM1: actin binding LIM protein 1; CBP: CREB binding protein; DKK3: dickkopf WNT signaling pathway inhibitor 3; DVL1: dishevelled 1; EGFR: epidermal growth factor receptor; EGLN2/PHD1: egl-9 family hypoxia inducible factor 2/prolyl hydroxylase 1; HIF2α: hypoxia-inducible factor 2-alpha; ID2: inhibitor of DNA binding 2; NFAT: nuclear factor of activated T-cells; RTKs: receptor tyrosine kinases and VEGFR2: vascular endothelial growth factor receptor 2. n.d.: not determined.

## Supplementary Materials

The following are available online at https://www.mdpi.com/2072-6694/12/8/2106/s1: Table S1. Differential expression of DYRK genes in TCGA tumor samples.
